# Optimization of Pectin-Zein Beads via Response Surface Methodology for Enhanced Colon-Targeted Delivery of *p*-Coumaric Acid from Rice Husk Extract

**DOI:** 10.3390/foods14122034

**Published:** 2025-06-09

**Authors:** Ilaria Frosi, Raffaella Colombo, Chiara Milanese, Adele Papetti

**Affiliations:** 1 Department of Drug Sciences, University of Pavia, 27100 Pavia, Italy; ilaria.frosi01@universitadipavia.it (I.F.); raffaella.colombo@unipv.it (R.C.); 2 Physical Chemistry Section, Department of Chemistry, University of Pavia & C.G.S.I., 27100 Pavia, Italy; chiara.milanese@unipv.it; 3 Department of Drug Sciences, University of Pavia & C.G.S.I., 27100 Pavia, Italy

**Keywords:** rice husk, polyphenols, pectin, zein, beads, encapsulation efficiency, simulated digestion, bioaccessibility

## Abstract

The generally very low bioaccessibility of polyphenols can be enhanced through several different strategies, especially when these metabolites are components of extracts used as food ingredients. This work explores the efficacy of pectin-zein beads as carriers for delivering *p*-coumaric acid), the main component of rice husk extract. Ten formulations were prepared using the ionic gelation technique, employing a Taghuci Design of Experiments to optimize zein, pectin, and CaCl_2_ concentrations. Zein content was found as the main parameter affecting the encapsulation efficiency. The highest value (51.77 ± 1.13%) was achieved using 10% zein, 3% pectin, and 4% CaCl_2_. *p*-coumaric acid bioaccessibility in the raw and encapsulated extracts was evaluated by adopting the Infogest digestion protocol and simulating a colon phase with Pectinex^®^ Ultra SPL enzymes, evidencing that pectin-zein beads effectively improved *p*-coumaric acid stability in the extract. The encapsulation highly preserves *p*-coumaric acid during the gastric phase (bioaccessibility index 34%); conversely, an increased release was registered at the intestinal level, reaching approximately 80% and 100% during the duodenal and colon steps, respectively. Therefore, pectin-zein beads were demonstrated to be a promising tool for the development of active ingredients suitable for functional foods/food supplements aimed at enhancing health benefits through controlled intestinal delivery of bioactives.

## 1. Introduction

Several different experimental studies have highlighted that polyphenols offer numerous health benefits [1], but their effectiveness is mainly hindered by several factors, such as the limited solubility and bioaccessibility in the gastrointestinal (GI) tract due to the pH value of this environment, their interaction with the food matrix, and the different occurring metabolic processes [2]. To address these issues, different technologies have been explored, with encapsulation emerging as a common method to enhance the stability and shelf life of phenolic compounds [3]. The most commonly used carriers for such systems are proteins, polysaccharides, lipids, or mixed systems, and the encapsulation mechanism can be based on different interaction patterns with polyphenols [4,5,6,7,8]. Polysaccharide-based carriers, mainly utilizing pectin and chitosan, can easily form porous and highly stable networks, and in recent years, the use of innovative carrier polymers derived from vegetable sources, particularly agricultural and food by-products, is gaining attention as a sustainable ingredient solution [9,10,11,12,13].

Pectin, a heteropolysaccharide mainly composed of galacturonic acid, has recently gained attention for its potential in circular economy practices because it is easily extracted from agricultural waste [14,15]. Anyway, it has been shown that the degree of esterification on galacturonic acid residues highly affects the physicochemical features and, therefore, the possible applications. In fact, low-methoxylated pectin (LMP) is more stable in an acidic medium than high-methoxylated pectin (HMP), which at low pH and in the presence of a high amount of sugars generates a gel through the formation of hydrogen bonds and hydrophobic interactions [16]. In recent years, many studies have indicated LMP as an effective carrier system, demonstrating high encapsulation efficiency (EE%) and loading capacity for delivering nutraceuticals [17,18]. Notably, pectin exhibits resistance to gastric conditions and human digestive enzymes, particularly to protease and amylase action [19], but can be fully digested by colon microflora. This unique property makes pectin a promising choice, and pectin-based formulations are suitable for colonic targeting and probiotics encapsulation [10,20]. Moreover, in pectin-based encapsulation systems, a controlled release of the encapsulated compounds has been demonstrated, enhancing their availability for intestinal absorption [21]. Polyphenol-loaded nanoparticles could be a suitable strategy to improve the stability of such biocompounds and target their release, as recently demonstrated for papaya, chokeberries, and pomegranate polyphenolic extracts [22,23,24]. Zein was also used in combination with pectin to form stable and resistant hydrogel beads for a colon-specific compound delivery [25,26].

Recently, our research team investigated the potential bioactivity of a rice husk extract (RHE) obtained by the rice by-products provided by a Lombard (Italian Region) organic farm. The RHE phytocomplex contained different flavonoids and hydroxycinnamic acids, among which *p*-coumaric acid (*p*-CA) was the most prominent and, therefore, selected as a marker compound. Different in vitro experiments indicated RHE as a promising candidate for its use in health-focused formulation, pending bioaccessibility studies [27]. *p*-CA has poor aqueous solubility and, therefore, low bioaccessibility, also being a potential substrate of p-glycoprotein (P-gp), an efflux-mediated transporter at the intestinal level. Currently, lipid-based carriers are the most investigated solution for poorly soluble compounds. In particular, microemulsion systems made of oil, water, surfactant, and co-surfactant are considered the most efficient tools to improve the stability and the intestinal absorption of *p*-CA and for the P-gp inhibition [28,29].

With this background, the main objective of this study was to assess the bioaccessibility of *p*-CA from RHE using the Infogest 2.0 protocol to simulate the in vitro digestion process. Additionally, we aimed to investigate the feasibility of a different approach to stabilize *p*-CA using pectin-zein complex hydrogel beads (never before used for *p*-CA) to encapsulate the extract, optimizing the experimental parameters affecting EE% by Design of Experiments (DOE) approach (Taguchi method). A pectin-zein-based system was selected both as it could be suitable to enhance RHE bioaccessibility and as it could represent a good solution for the colon-specific delivery of bioactives.

The obtained results (*p*-CA improved stability, with the release completed at the colon level in the simulated in vitro system) highlight the possibility of developing a colon-targeted delivery system for *p*-CA based on a polysaccharide carrier with important impact polyphenols’ bioactivity at the colon level.

## 2. Materials and Methods

### 2.1. Chemicals

Ethanol 96% (*v*/*v*), potassium chloride, potassium dihydrogen phosphate, sodium chloride, sodium hydrogen carbonate, ammonium carbonate, and sodium hydroxide were supplied by Carlo Erba (Milano, Italy). Methanol (ACS reagent, ≥99.8%; HPLC grade, ≥99.9%; and HPLC-MS hypergrade), formic acid (reagent grade, ≥95%, HPLC and HPLC-MS grade, 98–100%), *p*-coumaric acid (purity grade, ≥98.0%), hydrochloric acid 1 M, calcium chloride (CaCl_2_) dihydrate, magnesium chloride hexahydrate (purity grade, ≥99%), zein, type VI-B porcine pancreatic α-amylase, pepsin from porcine gastric mucosa (≥400 U/mg), bile extract porcine, pancreatin (8 × USP) from porcine pancreas, protease from Streptomyces griseus type XIV (≥3.5 U/mg), Pectinex^®^ Ultra SPL (aqueous solution ≥3800 units/mL) were provided by Merck KGaA (Darmstadt, Germany). Genu^®^Pectin LM 102 AS was provided by Azelis Group NV (Berchem, Belgium). Water was obtained from Millipore Direct-QTM system (Merk-Millipore, Milan, Italy).

### 2.2. Rice Husk Extract (RHE)

Rice husk was kindly provided by a local organic farm in Pavia zone (Lombardy region, Italy). The material underwent overnight (12 h) drying in an oven at 45 °C (until moisture reached approximately 1%) and was subsequently ground using a chopping knife and passed through a 500 μm sieve screen. Microwave-Assisted Extraction (MAE) process and hydroalcoholic solvent mixtures method were optimized to obtain the final rice husk extract (RHE), which was finally dried and chemically characterized by RP-HPLC-DAD-ESI-MS^n^. All the experimental details for preparation, extraction, and analysis procedures were reported in Frosi et al. [27].

### 2.3. RHE Encapsulation

#### 2.3.1. Experimental Design

The Taguchi Orthogonal Array was used to optimize the nanoformulation for RHE delivery. A full factorial array analysis was chosen for this purpose, as it allowed for a comprehensive exploration of factors without requiring excessive time in the results. In this DOE, a factorial design 3*3-1 with 9 experiments was conducted, involving the study of 3 factors (X_1_: pectin concentration; X_2_: zein concentration; X_3_: CaCl_2_ concentration) with each of them tested at 3 levels (−1; 0; +1) [30]. Additionally, two central point experiments were included, resulting in a total of 11 runs, as outlined in Table 1.

#### 2.3.2. Preparation of RHE Pectin-Zein Hydrogel Beads

Pectin-zein hydrogel beads were prepared according to the ionic gelation technique [24,31]. The RHE (2.5 mg/mL) was dissolved in a pectin solution (0.5%, 1.75%, and 3%, *w*/*v*) under continuous stirring (1000 rpm) for 30 min at room temperature (25 ± 0.5 °C). The sample solution was injected into a 75% ethanol solution containing zein and CaCl_2_ using a syringe (needle e.d. 0.91 mm, dripping time about 1 drop/5 s), and the mixture was continuously stirred. The considered zein concentration in the ethanol solution was 1%, 5.5%, or 10% (*w*/*v*), while CaCl_2_ concentrations were adjusted to 0.5%, 2.25%, and 4% (*w*/*v*). Discrete pectin droplets were formed. After preparation, these droplets underwent a 15 min curing process in the receiving phase and subsequently were washed twice with ethanol (96%, *v*/*v*) and Milli-Q water before undergoing freeze-drying (Modulyo freeze-drier s/n 5101, Akribis Scientific Ltd., Cheshire, UK). Ten different formulations were investigated using Response Surface Methodology (RSM) (see Section 2.3.1).

### 2.4. RP-HPLC-DAD Analysis and Method Validation

To quantify *p*-CA in RHE, an HPLC Agilent 1200 system (Waldbronn, Germany) equipped with an online degasser for the mobile phase, a quaternary pump, a diode array detector (DAD), and a Gemini^®^ C18 analytical column (150 × 2.0 mm i.d., 5 μm, Phenomenex, Torrance, CA, USA) was used. The system maintained a constant flow rate of 0.3 mL/min, with an injection volume of 20 μL. UV-Vis spectral data were acquired in the range of 200–700 nm, and chromatograms were recorded at 320 nm (characteristic wavelength for the hydroxycinnamic acids). Data acquisition and processing were performed using the ChemStation software B.04.01.

The mobile phase composition was binary: solvent A, consisting of 0.1% formic acid in water, and solvent B, consisting of methanol, were employed. A gradient elution was used for the separation, as follows: 0–10 min, 10–30% B; 10–20 min, 30–45% B; 20–30 min, 45–55% B; 30–35 min, 55–65% B; 35–40 min, 65–75% B; 40–45 min, 75–85% B; 45–48 min, 85% B, followed by a 12 min column reconditioning [27].

The analytical method was validated in accordance with the ICH guidelines for bioanalytical methods (ICH Steering Committee) [32]. Preliminary experiments were performed to assess matrix effect, using *p*-CA calibration curves constructed with *p*-CA (in the concentration range of 0.5–20 μg/mL) dissolved in rice husk extract (RHE) and in an aqueous methanol solution (50:50, *v*/*v*). Two distinct five-point calibration curves were obtained, with each point analyzed in triplicate. Chromatograms were recorded at 320 nm, and the injection volume was set at 10 μL.

Validation parameters included specificity (comparing chromatograms of *p*-CA with a blank solution), selectivity (comparing *p*-CA retention time in different solutions), linearity (constructing three *p*-CA calibration curves in the range of 0.5–20 μg/mL), intra-day precision (evaluating triplicate analyses for three consecutive days), accuracy (conducting recovery studies at three concentration levels), and determination of the limit of detection (LOD) and limit of quantification (LOQ) using signal-to-noise ratios of 3 and 10, respectively.

Preliminary analyses indicated the absence of matrix effect; hence, the external standard method was used for the validation procedure. All the analyses were performed by dissolving *p*-CA in a MeOH:H_2_O mixture, 50:50 *v*/*v*. To assess the method specificity, the *p*-CA (20 μg/mL) chromatogram was compared with that of a blank solution (mobile phase), and no other peaks were detected. Selectivity was confirmed by matching the retention time of *p*-CA standard and *p*-CA in RHE, registered at 320 nm. Over three separate days, a fresh calibration curve (5–20 μg/mL) was prepared and analyzed, and linear regression was performed using the least-squares method (Figure S1a,b). The average correlation coefficient of these three curves exceeded 0.9992, demonstrating good linearity. The method was accurate, with values ranging from 99.92 to 99.95%, and precise, considering that all standard deviation values were below 0.2%. Sensitivity was evaluated by determining the LOD and LOQ, which were found to be 0.009 μg/mL and 0.03 μg/mL, respectively. Considering the data, this analytical method is appropriate for quantifying *p*-CA in RHE.

### 2.5. Determination of Encapsulation Efficiency

The evaluation of RHE encapsulation efficiency (EE%) followed the methodology proposed by Contado et al. [33] with slight modifications. In brief, 10 mg of freeze-dried beads were suspended in 20 mL of methanol under stirring. After 10 min, 20 mL of an aqueous methanol solution (50:50, *v*/*v*) was added, and the mixture underwent magnetic stirring for 4 h, followed by an additional 1 h at 37 °C in a thermostatic bath. Subsequently, the solution was centrifuged at 5000 rpm for 20 min at 25 °C, and the supernatant was analyzed by HPLC, as outlined in Section 2.4.

*p*-CA was identified as the marker compound in the extract [27], and its EE (expressed as percentage) was determined using the following equation:

(1) EE (%) = ((Actual *p*-CA)/(Initial *p*-CA))*100

where Actual *p*-CA represents the amount of *p*-CA encapsulated in pectin-zein beads, and Initial *p*-CA is the amount of *p*-CA present in the RHE.

### 2.6. In Vitro Gastrointestinal Digestion Protocol

The stability assessment of both raw and encapsulated RHE was performed using a static in vitro simulated digestion method. The protocol is an improvement of the Infogest scheme and follows the established procedures detailed by Brodkorb et al. [34] for simulating oral, gastric, and intestinal digestion and by Zhang et al. [35] for colon digestion simulation.

During the simulated oral digestion phase, 2.5 mg/mL of the raw or the encapsulated (in pectin-zein beads) RHE was added to 4 mL of simulated salivary fluid (SSF) electrolyte stock solution. This mixture was then diluted to 10 mL using ultra-pure water, and 25 μL of CaCl_2_ (0.3 M) was added, followed by stirring through a magnetic stirrer for 2 min. Subsequently, 8 mL of simulated gastric fluid (SGF) electrolyte stock solution, 1.25 mL of pepsin solution (80 mg/mL in HCl 0.1 M), and 40 μL of CaCl_2_ were added. The pH of the gastric digestion mixture was adjusted to 3.0 ± 0.1 using HCl (1 M). Subsequently, the mixture was diluted to 20 mL with Milli-Q water and underwent incubation in a water bath shaker at 80 rpm for 2 h at 37 °C to simulate gastric digestion.

Keeping on the process, the gastric digestion mixture was supplemented with 16 mL of simulated intestinal fluid (SIF) electrolyte stock solution, incorporating 100 mg of porcine bile, 1.25 mL of pancreatin solution (32 mg/mL in water), and 5 μL of CaCl_2_. This mixture was then diluted to 40 mL using Milli-Q water. The pH was adjusted to 7.0 ± 0.1 using NaOH (1 M), and the mixture underwent incubation in a water bath shaker at 80 rpm for 2 h at 37 °C to simulate intestinal digestion.

For the colonic digestion phase simulation, the intestinal digestion mixture was combined with 2.5 mL of protease (1 mg/mL) and incubated for 1 h in a water shaker bath at 30 rpm. Following this step, 3 mL/L of Pectinex^®^ Ultra SPL (a blend mainly composed of pectintranseliminase, polygalacturonase, pectinesterase, and small amounts of hemicellulases and cellulase) was added, and the mixture was subjected to incubation in a water bath shaker at 30 rpm and 37 °C for 16 h.

At the end of each phase, the samples were heated in a water bath at 90 °C for 5 min to deactivate the enzymes and subsequently centrifuged at 5000 rpm for 10 min at 25 °C; the supernatant was freeze-dried, yielding a ready-to-use powder for bioaccessibility evaluation.

### 2.7. Bioaccessibility Evaluation

The quantification of *p*-CA in each digestion fraction for both raw RHE and encapsulated RHE was considered as an indicator of the sample portion potentially available for absorption. To evaluate bioaccessibility, the freeze-dried samples obtained post-digestion were reconstituted using an appropriate volume of 0.1% formic acid and methanol (50:50, *v*/*v*). Subsequently, the mixture was filtered on 0.45 µm RC syringe filters (Phenomenex, Torrance, CA, USA) before RP-HPLC analysis, following the validated method outlined in Section 2.4. The determination of the bioaccessibility index (BI) was performed considering the peak areas corresponding to *p*-CA. BI percentage was calculated as follows:

(2) BI (%) = ((soluble *p*-CA)/(initial *p*-CA))*100

where soluble *p*-CA indicates the concentration of *p*-CA in each digestion fraction for raw or encapsulated RHE, and initial *p*-CA indicates the concentration in the undigested raw or encapsulated RHE [36].

### 2.8. Statistical Analysis

The results were expressed as the mean ± standard deviation (SD) of the measurements obtained from at least three replicated experiments performed in duplicate. Differences were considered significant at *p* < 0.05 and *p* < 0.01. Statistical analysis was carried out using Microsoft Office 365. Statgraphics Centurion 19 software (Statgraphics Technologies, Inc., The Plains, VA, USA) was used for experimental design, model fitting, and data analysis. In particular, the analysis of the experimental results was performed by fitting them to the proposed equations using various statistical tools. The variability explained by the model was assessed through the coefficient of determination (R2), while the adjusted coefficient of determination (R2adj) was calculated to account for the number of variables included in the model. Analysis of variance (ANOVA) was employed to identify significant differences among the variables. Additionally, the impact of the independent variables on the response variables was visualized using contour and response plots created with the Statgraphics Centurion 19 software.

## 3. Results and Discussion

### 3.1. Optimization of the RHE Formulation

Polyphenols’ bioaccessibility and bioavailability are generally affected by the fluid composition of the GI tract. To address this challenge, it is common to use a carrier agent that stabilizes and facilitates the passage of polyphenols to the colon [3]. In this study, a commercial low-methoxyl (LMP) pectin from citrus peel was selected as a carrier for a preliminary investigation involving the selected RHE.

For RHE encapsulation, pectin-zein hydrogel beads were formulated using the ionic gelation technique [24,31]. Zein, a hydrophobic protein commonly found in corn kernels, was selected to be incorporated with pectin in the hydrogel bead formulation to enhance its stability, ensuring the targeted delivery to the small intestine. Zein plays a critical role in preventing premature release of the payload, which can occur when pectin swells upon contact with GI fluids. Additionally, pectin protects zein from protease digestion, preserving its integrity during gastric transit [37].

In a previous study, 2.5 mg/mL RHE was identified as the concentration possessing the highest bioactivity in in vitro antiglycative assays [27]; starting from this result, it was selected as the most suitable for encapsulation. To optimize the formulation, RSM was employed, focusing on exploring the effect of pectin, zein, and CaCl_2_ concentrations on EE%. The Taguchi orthogonal array was used for the experimental design due to its suitability for achieving consistent reductions in time and the number of experiments. It is noteworthy that orthogonal arrays are considered the optimal and widely used type of Taguchi array. Whenever feasible within time and budget constraints, the choice of orthogonal arrays in experiments is recommended. It typically involves striking a balance between the experiment’s cost, including the required time, and the desired precision of the results [30]. In our case, the preparation of pectin-based beads involves manual and time-consuming operations such as controlled stirring and dropwise formation using a syringe, making the execution of a large number of experiments particularly demanding. While the Box–Behnken Design (BBD) is a commonly used method for response surface analysis, it would require a substantially higher number of runs (typically ≥15 for three factors), significantly increasing the experimental workload. The Taguchi design, by contrast, allowed us to systematically investigate multiple parameters with only 11 well-structured experiments while maintaining orthogonality and statistical reliability. Therefore, this approach was not only practical but also well-suited to the experimental constraints of this study.

This DOE, specifically using an orthogonal array known as a factorial design 3*3-1, required nine experiments involving three levels (L), with each level tested three times [30]. Additionally, two central point experiments were included (runs No. 10 and 11). The selected ranges for experiments (scaled as −1 to +1) were as follows: pectin concentration (P) ranging from 0.5 to 3% *w*/*v*, zein concentration (Z) from 1 to 10% *w*/*v*, and CaCl_2_ concentration from 0.5 to 4% *w*/*v*. Table 2 summarizes the comprehensive experimental design, along with the observed EE%, calculated as reported in Section 2.5. The relative charts are reported in Figures S2–S4.

Ten different formulations were tested, with two replicates at the central point of experimentation.

The mathematical formula of the second-order polynomial equation derived from the response surface was as follows:

(3) EE (%) = −2.94695 + 26.7484*P + 1.53204*Z − 14.9672*CaCl_2_ − 7.04373*P^2^ − 1.21215*P*Z + 7.02705*P*CaCl_2_^2^ + 0.251235*Z^2^ + 1.71534*Z*CaCl_2_^2^ − 1.73741*CaCl_2_^2^

EE varied within the range of 4.54–61.54% for the different formulations. The analysis of variance (ANOVA) results, as detailed in Table 3, outlined the significant impact of zein concentration on RHE encapsulation (B:Z, *p* < 0.05).

**Figure 1:**
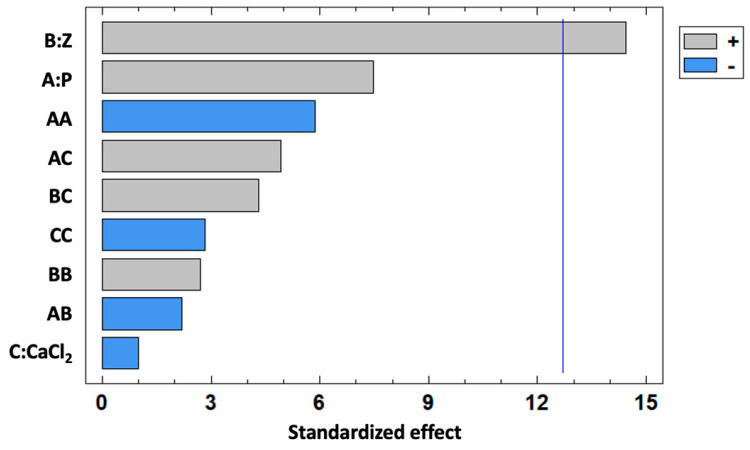
Standardized Pareto chart effect for *p*-coumaric acid (*p*-CA) encapsulation efficiency, considering the influence of zein (Z), pectin (P), and CaCl_2_ concentrations. The positive and negative effect of the factors (reported as horizontal bars) on the response variable (EE%) is represented with gray and blue colors, respectively. The vertical line tested the significance of the effect at the 95% confidence level.

This outcome was visually represented in the Pareto chart (Figure 1), which showed the positive effect of increasing zein concentration on EE% (calculated as reported in Section 2.5).

These observations aligned with the findings of Liu et al. [31], who developed pectin-zein beads for the colon delivery of indomethacin (I) and bovine serum albumin (BSA). Liu et al. [31] demonstrated low EE% for pectin beads (I = 180 ± 21 mg; BSA = 150 ± 9 mg) but observed a significant improvement with increasing zein concentrations (maximum values: I = 223 ± 11 mg for 5% *w*/*v* of zein; BSA = 210 ± 14 mg for 10% *w*/*v* of zein). Similar results were reported by Mukhidinov et al. [38] in the encapsulation of piroxicam in zein-pectin hydrogel microspheres. Once again, the zein content played a pivotal role, with minimal encapsulation in the absence of this compound (1:1 ratio Z/P, 37% of piroxicam in the complex) and a noteworthy increase in encapsulation extent as the zein fraction in the complex was raised (5:1 ratio Z/P, 93.9% of piroxicam encapsulated). Conversely, Chotiko et al. [39] used zein to stabilize a pectin–whey protein complex encapsulating anthocyanins extracted from rice bran. They tested different carrier agents and combinations to develop an intestinal-targeted formulation, i.e., pectin alone (P), pectin combined with zein (P+Z), or whey protein (P+WP), and a ternary mixture combining pectin, whey protein, and zein (P+WP+Z). P+WP and P+WP+Z showed controlled and slow release at the intestinal level compared to the other formulations. Therefore, the addition of zein did not significantly affect the formulation, probably because of the insufficient zein content.

Regarding the other selected parameters, pectin and CaCl_2_ concentrations had no significant effect on EE%, but they had an opposite influence on *p*-CA encapsulation (a positive and a negative impact, respectively) (Figure 1). As regards the Z/P ratio set at 3.33:1, pectin (3% *w*/*v*) was an effective thickening and stabilizing agent in bead preparation, suppressing swelling that could cause a premature *p*-CA release. Similarly, a high concentration of CaCl_2_ (4% *w*/*v*) contributed to a dissolution-control pattern for *p*-CA-release.

Consequently, the optimal conditions for maximizing EE% were identified as follows: 3% *w*/*v* of P, 10% *w*/*v* of Z, and 4% *w*/*v* of CaCl_2_. Under these conditions, the calculated EE reached an impressive 83.2%, with a 95% confidence interval for the mean value covering from 43.0% to 122.0%. To validate the precision of our model, three additional experiments were performed under these optimally predicted conditions, resulting in a mean EE of 51.77 ± 1.13%. This outcome indicated that EE% value is in the confidence interval range and our fitted model is reliable.

### 3.2. Bioaccessibility of p-Coumaric Acid in Raw and Encapsulated RHE

The raw and encapsulated extracts (2.5 mg/mL) underwent digestion using the Infogest digestion protocol 2.0, which simulates the in vitro oral, gastric, and intestinal phases of the digestion process using a blend of electrolytes and enzymes. An additional colon phase, incorporating Pectinex^®^ Ultra SPL which is a mixture of enzymes able to degrade the pectin polymer, was included following the method proposed by Zhang et al. [35].

**Figure 2:**
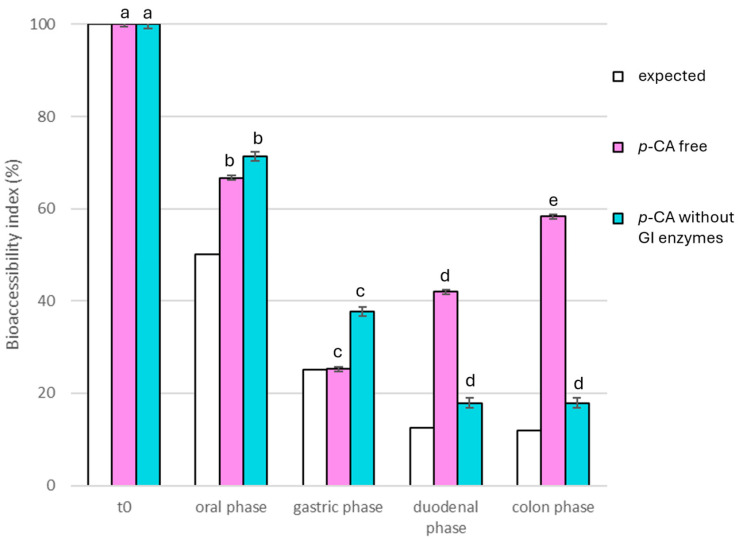
The bioaccessibility index of *p*-coumaric acid (*p*-CA) under the gastrointestinal tract is illustrated. The white bars represent the concentration of the theoretically expected *p*-CA (obtained by the gradual dilution factor occurring during the process); the light blue bars show the concentration of *p*-CA detected at different digestion steps without the effect of the gastrointestinal enzymes; the violet bars indicate the *p*-CA concentration in the different RHE-digested fractions. Different superscript letters for each sample series indicated significant differences (*p* < 0.05) among the different digestion phases.

For the digestion of the raw extract, 12.5 mg of RHE was solubilized in 5 mL of water (according to the Infogest protocol fixed sample volume). Conversely, for the encapsulated extract, 5 mL of a 2.5 mg/mL solution of RHE was loaded into the beads. The resulting formulation was freeze-dried and then added to 5 mL of water before the simulated digestion process. The mixtures obtained at the end of each digestion phase were heated to 90 °C for 5 min to deactivate the GI enzymes and centrifuged; the supernatants were freeze-dried. Bioaccessibility was calculated by comparing the concentration of *p*-CA before and after the digestion step and the results were reported in Figure 2.

Additionally, the digestion protocol was applied to the raw extract without the addition of enzymes, but by simply adding electrolyte aqueous solutions (*p*-Ca without GI enzymes) or to the extract submitted to the simulated GI fluids (constituted by proper electrolytes solution and enzymes) (*p*-Ca free) to estimate the putative effect of the enzymes on RHE digestion. Figure 2 illustrates the instability of the extract, already evident at the oral phase and particularly strong at the intestinal level. In fact, *p*-CA free concentration notably increased compared to the expected theoretical concentration (white bars) based on the dilution factor of the extract through the digestion process. Differently, in *p*-CA-free samples at the duodenal and colon levels, about 42% and 58% of the initial *p*-CA concentration was detected, respectively, instead of about the expected 12.5% and 12%.

This result was likely due to the presence of *p*-CA linked to protein or polysaccharide components, subsequently released during the GI process through enzyme action. A similar behavior was reported in the literature by Massarioli et al. [40] for the bioaccessibility of *p*-CA in a peanut extract. In fact, after the digestion of the extract by the Infogest protocol, free *p*-CA, differently from coumaroyl-derivatives, exhibited a significant increase at the intestinal level (*p* < 0.05), with the highest bioaccessibility (around 370%). These remarkable results aligned with and supported our findings, as esterases in the small intestine and colon can cleave ester bonds of esterified hydroxycinnamic acids, releasing *p*-CA in free form into the lumen [40].

**Figure 3:**
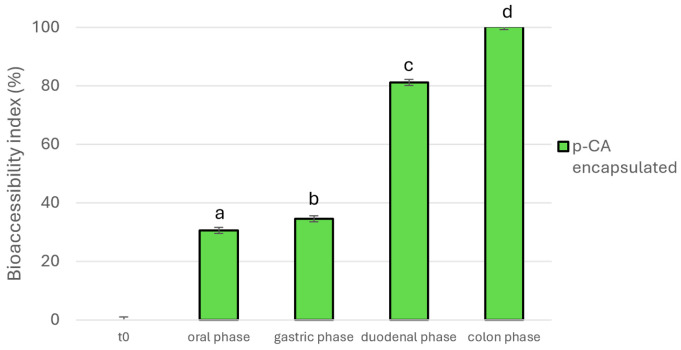
Bioaccessibility index of encapsulated *p*-coumaric acid (*p*-CA) under the gastrointestinal tract. Different superscript letters indicated significant differences (*p* < 0.05) among the different digestion phases.

Subsequently, we assessed the *p*-CA bioaccessibility in the encapsulated extract, and the *p*-CA release profile is reported in Figure 3. After encapsulation, approximately 30% of *p*-CA was released within the initial 2 h of digestion under the gastric acidic conditions, followed by an increase to about 80% during the duodenal phase, reaching a complete release at the colon level. These results indicate the successful development of an intestinal-targeted formulation, which enhanced the colon-targeted delivery of *p*-CA from RHE. In fact, the formulation was able to protect the *p*-CA release in the first steps of digestion by reducing its release at oral and gastric level and increasing it at intestinal level.

Notably, at the end of the digestion, the total amount of *p*-CA present in the raw extract was released from the beads. However, these empirical findings contradicted the EE% calculated for the optimal formulation, which was approximately 50%. This discrepancy was attributed to the partial efficiency of the method used for determining the encapsulation efficiency and the assumption of an incomplete release of *p*-CA from the formulation, resulting in an underestimation of its concentration. The EE% determination method used in this research was based on Contado’s et al. [33] approach, with slight modifications. Contado et al. [33] compared two protocols for assessing the concentration of resveratrol loaded in eudragit pectin-zein nanoparticles. The first one involved the use of DMSO as a solvent to dissolve the particles, with UV-Vis measurements for concentration assessment; conversely, the second strategy involved the use of an aqueous methanolic solution (50:50 *v*/*v*) and HPLC measurements. Concerning our study, we used the second protocol due to its practicality and the availability of a validated HPLC-DAD method for determining *p*-CA concentration. However, as stated by Contado et al. [33], this method yielded EE% lower than those obtained with UV-Vis measurements, likely because DMSO was more effective at dissolving all the particles. As a matter of fact, particles were not completely dissolved, requiring filtration before HPLC injection, thereby limiting their quantification. Similarly, we hypothesized that our beads were not fully dissolved in the aqueous methanolic solution, potentially leading to an underestimation of the extract encapsulation. After digestion, the observed complete recovery of *p*-CA was probably due to the action, at the colon level, of pectinases, which efficiently broke down the beads and facilitated the complete release of *p*-CA (BI = 100.2 ± 6.60%).

In the literature, RHE- or *p*-CA-based formulations are mainly aimed at topic applications, and nanostructured lipids are the most used carriers [41,42]. As regards the oral formulations, oil in water microemulsion systems have been proposed to promote *p*-CA bioaccessibility [29]. Different combinations of oil, surfactant/co-surfactant, and water were investigated, and two formulations (Labrasol-Tween 80-water-Labrafil M1944 CS and Labrasol-Tween 80-water-Capryol 90-Transcutol P) were tested in rat model by monitoring *p*-CA plasma concentration. Both highlighted the increase in *p*-CA absorption with a maximum plasma concentration reached in 1 h followed by a decrease over time (8 h), indicating a strong and rapid absorption at the gastrointestinal level without a potential colon-targeted delivery.

Polymer-based beads represent a good alternative to encapsulate phenolic compounds (such as *p*-CA, tannic acid, chlorogenic acid, gallic acid, caffeic acid, naringin, and hesperidin). Calcium alginate beads prepared via an ionic gelation technique using different copolymers (gum arabic, K-carrageenan, guar gum) have been recently tested by Demircan et al. [43]. The highest *p*-CA EE was obtained for guar gum, with a value of 39.52 ± 1.53%, which is lower than that obtained for pectin-zein beads herein proposed. In addition, a comparison of the bioaccessibility index was not possible as no digestion study was performed. Chitosan-loaded *p*-CA nanoparticles are the most used formulation, and it was widely investigated in different in vitro and in vivo studies. These systems highlighted the protective effect exerted on *p*-CA and, therefore, on its bioactivities (i.e., antioxidant and anti-inflammatory) [44,45]. No investigation about the effect of a simulated digestion process has been reported before the in vivo studies.

## 4. Conclusions

This study demonstrates the potential of pectin-zein beads as effective delivery systems for the intestinal targeting (more specifically for colon-targeted delivery) of bioactive compounds, as evident by the successful encapsulation of a rice husk polyphenolic extract and its subsequent release profile under simulated GI conditions. This investigation highly contributes to elucidating the role of zein in the release profile of encapsulated extracts; such encapsulation significantly improves the stability of the extract during digestion, preventing the early release of *p*-CA and ensuring a controlled release throughout the digestive process.

However, discrepancies between the encapsulation efficiency and the post-digestion recovery of the marker compound highlight the need for more accurate, reliable, and robust methods for assessing the encapsulation efficiency and accurately quantifying the bioactive compound release from such formulations.

Future perspectives could deal with the improvement of the proposed encapsulation technique, optimizing strategies and performing mechanistic insights into the release kinetics of encapsulated compounds to obtain even more effective and specific systems for *p*-CA delivery.

In conclusion, this study underlines the real possibility to re-cycle agricultural by-products to give them a second life as suitable functional ingredients for the food and food supplements industries, using a green and sustainable approach. This could contribute to the fulfillment of the 2030 Agenda for Sustainable Development goals. In addition, the evaluation of *p*-CA bioaccessibility by the in vitro digestion simulation supported the efficacy of the proposed colon-targeted delivery system. Finally, pectin-zein-based beads could be successfully used in different formulations (such as capsules or granulated forms) for food supplements or functional foods (such as beverages). In fact, this technique is cost-effective and easily automatized and scaled up, making the procedure more rapid and reproducible.

## Figures and Tables

**Table 1:** Factorial design for the optimization of the pectin-zein beads. Coded variables.

Run (No.)	X_1_	X_2_	X_3_
1	−1	−1	−1
2	0	−1	0
3	+1	−1	+1
4	−1	0	0
5	0	0	+1
6	+1	0	−1
7	−1	+1	+1
8	0	+1	−1
9	+1	+1	0
10	0	0	0
11	0	0	0

**Table 2:** Taguchi orthogonal array for the optimization of pectin-zein hydrogel beads. Actual variables.

Run (No.)	P (% *w*/*v*)	Z (% *w*/*v*)	CaCl_2_ (% *w*/*v*)	EE (%)
1	0.50	1.0	0.50	4.54
2	1.75	1.0	2.25	11.01
3	3.00	1.0	4.00	15.57
4	0.50	5.5	2.25	8.02
5	1.75	5.5	4.00	25.91
6	3.00	5.5	0.50	17.27
7	0.50	10.0	4.00	38.05
8	1.75	10.0	0.50	48.33
9	3.00	10.0	2.25	61.54
10	1.75	5.5	2.25	31.27
11	1.75	5.5	2.25	34.88

**Table 3:** Analysis of variance of the orthogonal array model for the optimization of pectin-zein beads.

Source	Sum of Squares	df	Mean Square	F Ratio	*p* Value
A:P	364.39	1	364.39	55.92	0.0846
B:Z	1361.09	1	1361.09	208.88	0.0440
C:CaCl_2_	6.28	1	6.28	0.96	0.5059
AA	223.62	1	223.62	34.32	0.1076
AB	30.99	1	30.99	4.76	0.2737
AC	157.52	1	157.52	24.17	0.1277
BB	47.78	1	47.78	7.33	0.2252
BC	121.65	1	121.65	18.67	0.1448
CC	52.27	1	52.27	8.02	0.2161
Total error	6.52	1	6.52		

## Data Availability

The original contributions presented in this study are included in the article/Supplementary Materials. Further inquiries can be directed to the corresponding author.

## Supplementary Materials

The following supporting information can be downloaded at https://www.mdpi.com/article/10.3390/foods14122034/s1, Figure S1. Calibration curves and corresponding equations of *p*-CA in (a) an aqueous methanol solution (50:50, *v*/*v*) and (b) in RHE. Please note that the standard deviations are expressed as vertical error bars in black on each point. Figure S2. The average response plot of Z%. Figure S3. The average response plot of P%. Figure S4. The average response plot of CaCl_2_%.

## Abbreviations

The following abbreviations are used in this manuscript:

ANOVA	Analysis of variance
*p*-CA	*p*-coumaric acid
DOE	Design of Experiments
LMP	Low-methoxylated pectin
HMP	High-methoxylated pectin
RHE	Rice husk extract
MAE	Microwave-Assisted Extraction
RSM	Response Surface Methodology
ICH	International Council for Harmonisation
LOD	Limit of detection
LOQ	Limit of quantification
SSF	Simulated salivary fluid
SGF	Simulated gastric fluid
SIF	Simulated intestinal fluid
BSA	Bovine serum albumin
GI	Gastro-intestinal
EE	Encapsulation efficiency
