# Supplementary material for: Optimization of Pectin-Zein Beads via Response Surface Methodology for Enhanced Colon-Targeted Delivery of p-Coumaric Acid from Rice Husk Extract
Source: Foods. 2025 Jun 9;14(12):2034. doi: 10.3390/foods14122034 (PMC12191771; doi:10.3390/foods14122034)
Supplement: Supplementary file 1 [file foods-14-02034-s001.zip › foods-3618711-supplementary.pdf]

Supplementary material

(a)

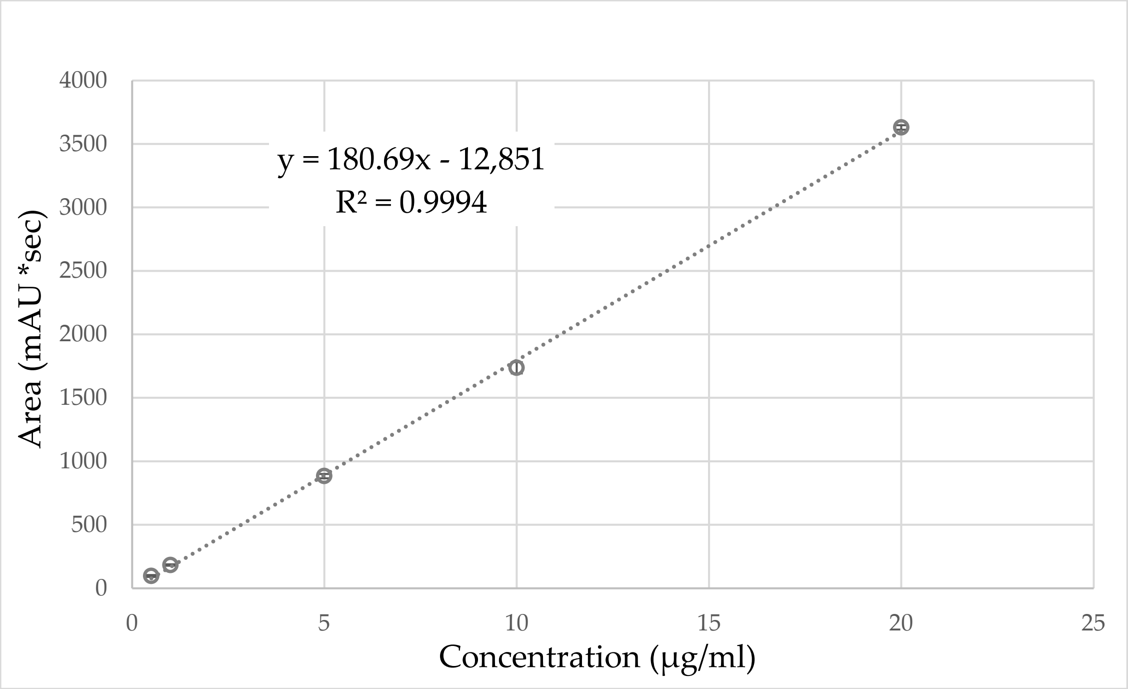

(b)

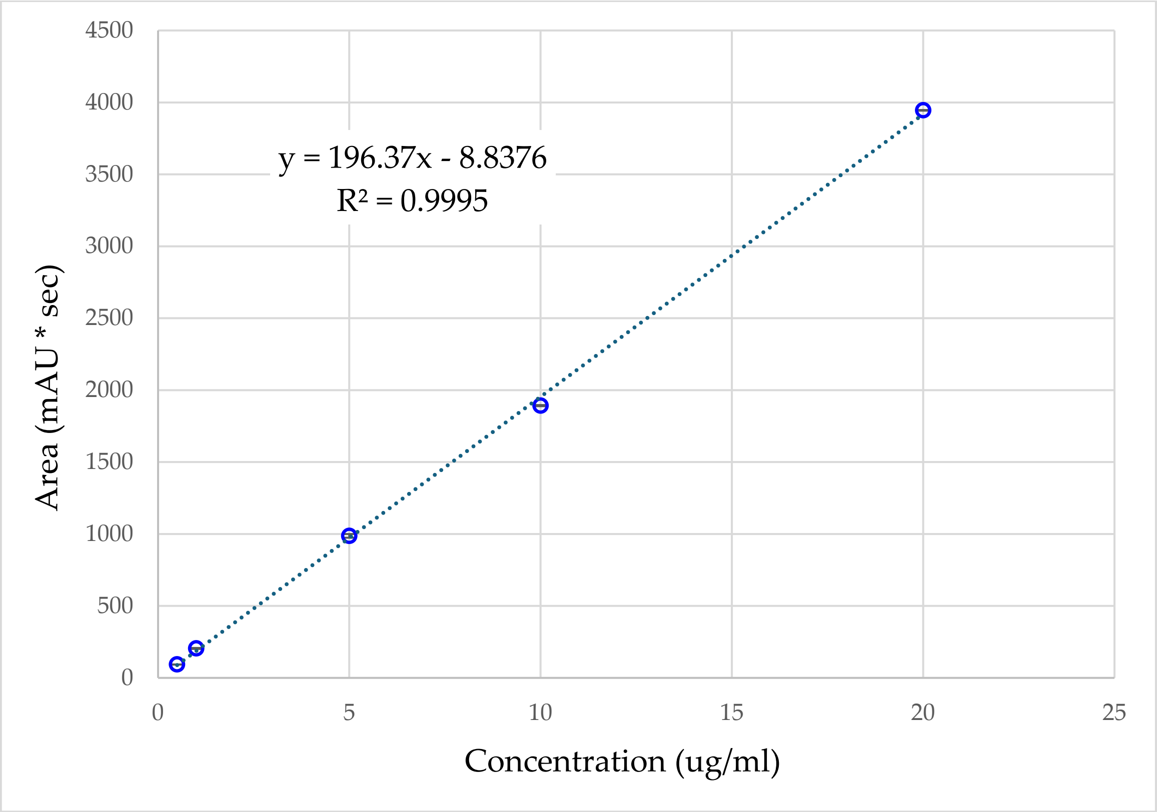

Figure S1. Calibration curves and corresponding equations of *p*-CA in: (a) an aqueous methanol solution (50:50, *v*/*v*) and (b) in RHE. Please note that the standard deviations are expressed as vertical error bars in black on each point.

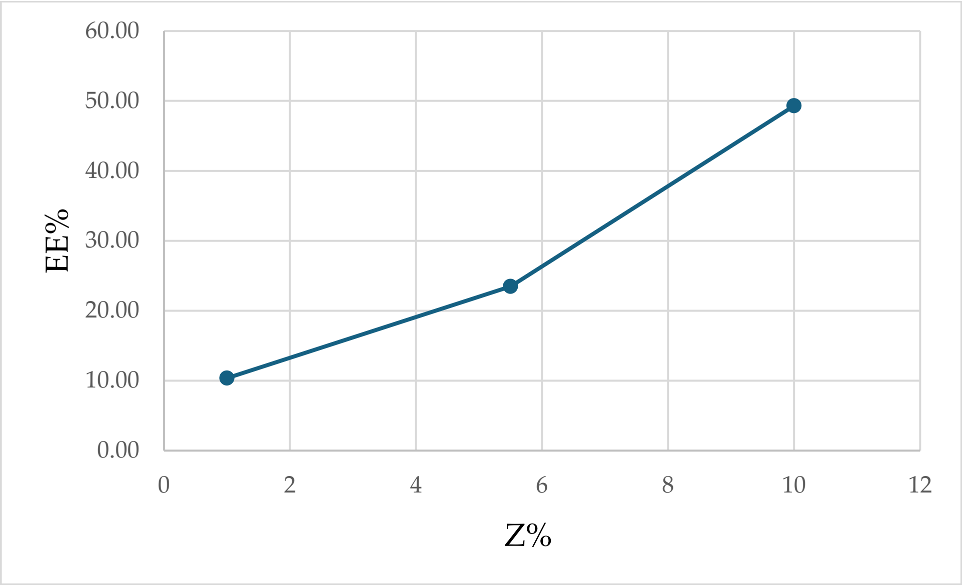

Figure S2. The average response plot of Z%.

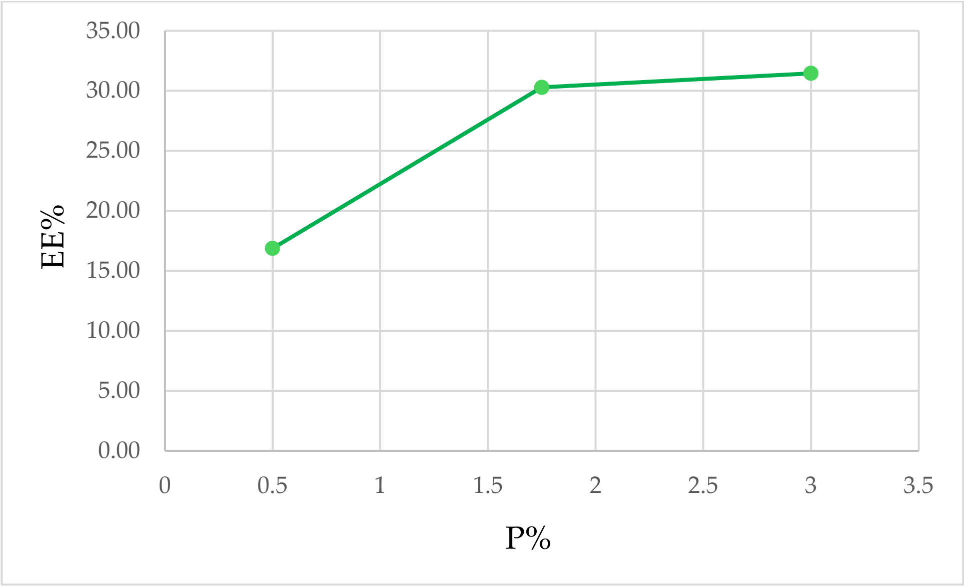

Figure S3. The average response plot of P%.

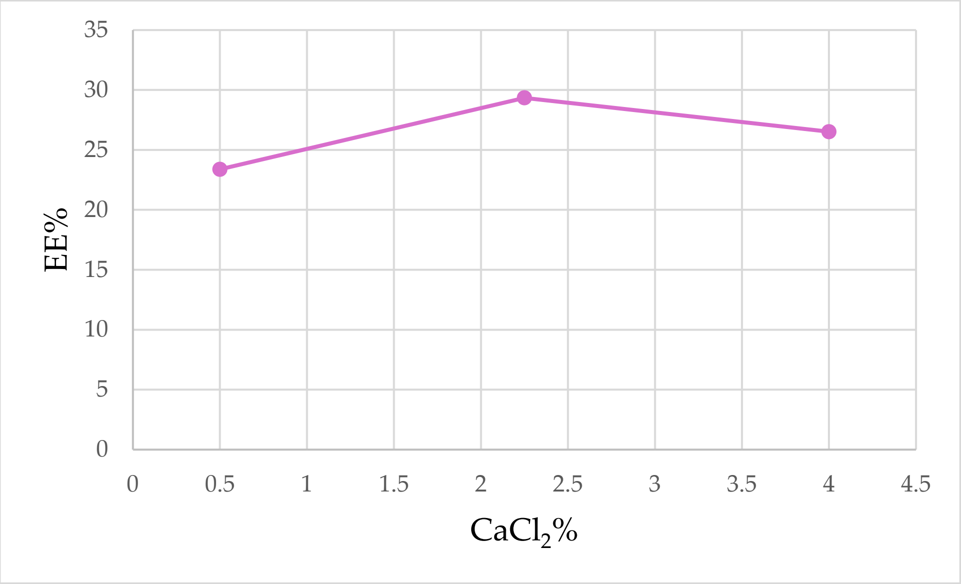

Figure S4. The average response plot of $CaCl_2$%.
